# Prognostic factors for abatacept retention in patients who received at least one prior biologic agent: an interim analysis from the observational, prospective ACTION study

**DOI:** 10.1186/s12891-015-0636-9

**Published:** 2015-07-30

**Authors:** Hubert G. Nüßlein, Rieke Alten, Mauro Galeazzi, Hanns-Martin Lorenz, Michael T. Nurmohamed, William G. Bensen, Gerd R. Burmester, Hans-Hartmut Peter, Karel Pavelka, Melanie Chartier, Coralie Poncet, Christiane Rauch, Manuela Le Bars

**Affiliations:** University of Erlangen-Nuremberg, Rheumatologische Schwerpunkpraxis, Kontumazgarten 4, 90429 Nuremberg, Germany; Schlosspark-Klinik University Medicine, Berlin, Germany; University of Siena, Siena, Italy; University Hospital, Heidelberg, Germany; VU University Medical Center/Jan van Breeman Research Institute, Amsterdam, Netherlands; St Joseph’s Hospital/McMaster University, Ontario, Canada; Charité-Universitätsmedizin, Berlin, Germany; University Medical Center Freiburg, Freiburg, Germany; Institute of Rheumatology and Clinic of Rheumatology, Charles University, Prague, Czech Republic; Chiltern International, Neuilly, France; Docs International, Nanterre, France; Bristol‐Myers Squibb, Munich, Germany; Bristol-Myers Squibb, Rueil-Malmaison, France

**Keywords:** Abatacept, Biologic agents, Cyclic citrullinated peptide, Heart failure, Prognostic factors, Multivariate analysis, Retention, Rheumatoid arthritis

## Abstract

**Background:**

The emergence of new therapies for the treatment of rheumatoid arthritis (RA), the paucity of head-to-head studies, and the heterogeneous nature of responses to current biologics highlight the need for the identification of prognostic factors for treatment response and retention in clinical practice. Prognostic factors for patient retention have not been explored thoroughly despite data for abatacept and other biologics being available from national registries. Real-world data from the ACTION study may supplement the findings of randomized controlled trials and show how abatacept is used in clinical practice. The aim of this interim analysis was to identify prognostic factors for abatacept retention in patients with RA who received at least one prior biologic agent.

**Methods:**

A large, international, non-interventional cohort of patients with moderate-to-severe RA who initiated intravenous abatacept in Canada and Europe (May 2008–January 2011) enrolled in the ACTION study. Potential prognostic factors for retention in this interim analysis (data cut-off February 2012; including patients from Canada, Germany, Greece, and Italy) were baseline demographics and disease characteristics, medical history, and previous and concomitant medication. Clinically relevant variables with *p* ≤ 0.20 in univariate analysis and no collinearity were entered into a Cox proportional hazards regression model, adjusted for clustered data. Variables with *p* ≤ 0.10 were retained in the final model (backward selection).

**Results:**

The multivariate model included 834 patients. Anti-cyclic citrullinated peptide (CCP) antibody positivity (hazard ratio [95 % confidence interval]: 0.55 [0.40, 0.75], *p* < 0.001), failure of <2 prior anti-tumor necrosis factors (TNFs) (0.71 [0.56, 0.90], *p* = 0.005 versus ≥2 prior anti-TNFs), and cardiovascular comorbidity at abatacept initiation (0.48 [0.28, 0.83], *p* = 0.009) were associated with lower risk of abatacept discontinuation. Patients in Greece and Italy were less likely to discontinue abatacept than patients in Germany and Canada (Greece: 0.30 [0.16, 0.58]; Italy: 0.50 [0.33, 0.76]; Canada: 1.04 [0.78, 1.40], *p* < 0.001 versus Germany).

**Conclusions:**

Real-world prognostic factors for abatacept retention include anti-CCP positivity and fewer prior anti-TNF failures. Differences in retention rates between countries may reflect differences in healthcare systems. The finding that abatacept has potential advantages in patients with cardiovascular comorbidities needs to be confirmed in further research.

**Electronic supplementary material:**

The online version of this article (doi:10.1186/s12891-015-0636-9) contains supplementary material, which is available to authorized users.

## Background

Abatacept is a fully humanized fusion protein that acts as a selective T-cell co-stimulation modulator. It is approved globally for the treatment of moderate-to-severe rheumatoid arthritis (RA) in patients with an inadequate response to one or more disease-modifying anti-rheumatic drugs (DMARDs; including methotrexate [MTX] or an anti-tumor necrosis factor [TNF]) [1–4]. In Canada, abatacept can also be used in combination with MTX for the treatment of moderate-to-severe RA in patients who are MTX-naïve [1–4]. Abatacept is available in subcutaneous and intravenous (IV) formulations [1–4], and a favorable efficacy and safety profile has been demonstrated in randomized controlled trials [5, 6]. IV abatacept received regulatory approval for the treatment of RA in 2006 in Canada and in 2007 in Europe. ACTION (AbataCepT In rOutiNe clinical practice) is a large, real-world, prospective, observational cohort study of patients with RA from Europe and Canada who were receiving IV abatacept and were followed for a maximum of 2 years. Results from a 6-month interim analysis of the ACTION study suggest that abatacept is an effective and well-tolerated treatment option in patients with RA [7].

The emergence of new therapies for the treatment of RA, the paucity of head-to-head studies, and the heterogeneous nature of responses to current biologics highlight the need for the identification of prognostic factors for treatment response and retention in clinical practice. The identification of prognostic factors may support individualized treatment strategies and could aid physicians in making informed therapeutic decisions [8].

Real-world studies may include patients with a wide range of disease activity levels and encompass regional variations in treatment, and can therefore supplement the findings of randomized controlled trials with strict inclusion and exclusion criteria [9]. Although some studies have identified prognostic factors for response to abatacept, few have been confirmed [10]. In the French Orencia and Rheumatoid Arthritis (ORA) registry, positivity for anti-cyclic citrullinated peptide (CCP) antibodies was associated with European League Against Rheumatism (EULAR) response in a multivariate analysis, after adjustment for Disease Activity Score (DAS)28 [11]. In an Italian study of 32 patients with RA who had been treated with abatacept, low levels of CD4 + CD28− and CD8 + CD28− T cells were associated with a greater likelihood of achieving remission at 6 months [12].

Treatment discontinuation can result from safety concerns or a lack of efficacy, but prognostic factors for patient retention have not been explored thoroughly despite data for abatacept and other biologics being available from national registries. In patients within the ORA registry, anti-CCP positivity occurred more frequently in patients who had continued abatacept treatment after 6 months, compared with patients who had discontinued abatacept (72.5 % versus 62.4 %, *p* = 0.02) [11]. In the Swedish Rheumatology register, 6-month abatacept retention rates were higher in patients who were biologic naïve compared with patients who had received 1 or ≥2 previous biologics (94 % versus 78 % and 77 %, respectively) [13]. In a pooled analysis of eight European RA registries, abatacept retention was strongly influenced by the number of previous biologics. Compared with patients who had not previously received biologic agents, retention was lower in patients who had received prior biologics (*p* < 0.01) [14].

This article reports prognostic factors for abatacept retention in patients with RA who had failed at least one prior biologic agent which were identified in an interim analysis from the international, non-interventional ACTION cohort study.

## Methods

### Study design and patient population

ACTION was a non-interventional, international, multicenter, cohort study that evaluated retention and effectiveness in patients with RA who had initiated IV abatacept in Europe (according to the abatacept Summary of Product Characteristics) and Canada (according to the abatacept Product Monograph) [2–4]. Patients were enrolled prospectively, within 3 months of abatacept initiation, between May 2008 and January 2011, and were followed for up to 2 years or, if the patient discontinued abatacept treatment before the 2-year endpoint, for up to 6 months after abatacept discontinuation. A detailed description of the ACTION study design has been reported previously in the context of a 6-month interim analysis [7, 15].

The study enrolled adult patients who were abatacept naïve, with established moderate-to-severe RA according to American College of Rheumatology revised criteria (1987) [16]. ACTION was conducted in accordance with the Declaration of Helsinki [17], the International Conference on Harmonization Good Clinical Practice Guidelines [18], and the Good Epidemiological Practice Guideline [19]. The study protocol and patient enrollment were approved by local ethics committees and regulatory agencies in accordance with each country’s requirements (first approval received on 31 January 2008, in Munich, Germany; Additional file 1: Table S1). Enrolled patients provided written informed consent.

Here we present an interim analysis of the ACTION study in patients who were treated in four countries (Canada, Germany, Greece, and Italy). All data received up to February 2012 were considered. The analysis included only patients who had received at least one prior biologic agent (the number of patients who had received no prior biologics was insufficient to build a robust model) and who had a baseline clinical assessment no later than 8 days after the first administration of abatacept [20].

### Definition of abatacept exposure

The dates of first and last abatacept infusions were collected, and abatacept discontinuation and reasons for discontinuation were reported by the rheumatologist at any follow-up visit. Exposure to abatacept was defined as the difference between the first and last date of abatacept infusion, plus 30 days. Patients who did not discontinue abatacept were censored at the time of last available data.

### Prognostic factors of retention

Data concerning potential prognostic factors, including known risk factors and clinically relevant variables, were collected at abatacept initiation. Continuous and categorized variables were considered in this analysis. Categorizations were based on validated cut-offs when available or on clinical expertise, previous literature, or descriptive statistics such as medians or quartiles. Sociodemographic variables were: country, age (continuous and <65 years, ≥65 years), sex, and body mass index (continuous and <25 kg/m^2^ [underweight/normal], 25– < 30 kg/m^2^ [overweight], 30– < 35 kg/m^2^ [obese class I], and ≥35 kg/m^2^ [obese class II/III]) [21]. Disease characteristics considered were: disease duration (continuous and ≤2 years, 3–5 years, 6–10 years, and >10 years), tender joint count (continuous out of a total score of 28), swollen joint count (continuous out of a total score of 28), C-reactive protein (CRP) quartile (<4 mg/L, 4– < 10 mg/L, 10– < 26 mg/L, and ≥26 mg/L), patient global assessment (continuous and based on median), physician global assessment (continuous and based on median), pain (continuous and based on median), DAS28 (erythrocyte sedimentation rate or CRP) (continuous and remission or low DAS [≤3.2], moderate DAS [>3.2–5.1], and high DAS [>5.1]), calculated Clinical Disease Activity Index (continuous and remission, low or moderate DAS [≤22] and high DAS [>22]), radiographic erosion, rheumatoid factor (RF) status, anti-CCP status, and RF and anti-CCP double-positive status. RF and anti-CCP peptide antibody status were based on the assessment at abatacept initiation or any assessment made previously. Comorbidity data (including cardiovascular comorbidity) were collected by assessment of medical history at abatacept initiation and were classified by System Organ Class (SOC) as per the Medical Dictionary for Regulatory Activities. All SOCs were described and considered. Also considered were: number of prior conventional (c)DMARDs (0–3, >3), number of prior anti-TNF agents (<2, ≥2), type of biologic agent received before abatacept (anti-TNF, other mechanism of action), and reason for discontinuation of last biologic agent (intolerance, primary inefficacy, secondary inefficacy, improvement in disease, and other reasons pooled together). Final considerations were: abatacept treatment pattern at initiation (monotherapy, combination with MTX, combination with other cDMARDs), prescription for MTX at initiation, and corticosteroid use before and at abatacept initiation (no corticosteroids, continuous use of corticosteroids, corticosteroids stopped at abatacept initiation, corticosteroids introduced at abatacept initiation).

### Statistical analysis

Abatacept retention rates and 95 % confidence intervals (CI) were described using Kaplan–Meier estimators. Overall retention rate and discontinuation for inefficacy and intolerance were presented at 12 months. Descriptive analysis was performed for all potential prognostic factors. All potential prognostic factors were tested in univariate analysis. For variables in which the categories ‘not available’ or ‘not done’ were predefined, univariate analyses were performed with and without consideration of this category; to be considered in the multivariate model both analyses were required to show consistent significant results. Clinically relevant variables, known risk factors, and prognostic factors with *p* ≤ 0.20 and no collinearity (two variables were collinear if *p* < 0.05 and V-Cramer >0.5) were retained in the multivariate model. Known risk factors at treatment initiation, such as infections and infestations, chronic obstructive pulmonary disease (COPD), diabetes mellitus, and tobacco use, were considered in the multivariate model regardless of results in the univariate analysis. Prognostic factors of abatacept retention were identified over the whole period until the data cut-off (February 2012) by multivariate analysis using a Cox proportional hazards model with clustered data (sandwich method) to account for dependence among patients from a single site. Prognostic factors with *p* > 0.10 were removed by backward selection. Interactions between the retained prognostic factors were tested and included in the final model if significant (*p* < 0.10). Univariate analyses were re-run for the final patient sample included in the final multivariate model. Results were presented as hazard ratios (HRs) with 95 % CIs and *p*-values. An HR >1 indicates higher likelihood of abatacept discontinuation, while an HR <1 indicates a lower likelihood of abatacept discontinuation. HRs are significant when the 95 % CIs do not overlap 1. A sensitivity analysis was performed, including all variables that were significant in the univariate analysis with no variable selection in the multivariate step.

## Results

### Patient population

Between May 2008 and January 2011, 1138 patients were enrolled in the ACTION study, 999 (87.8 %) of whom were enrolled in Canada, Germany, Greece, and Italy. A total of 865/999 (86.6 %) patients had received at least one prior biologic agent. Patient baseline demographic and disease characteristics and comorbidities are shown in Table 1. Mean (standard deviation [SD]) age was 56.5 (12.1) years, 83.1 % of patients were women, mean (SD) RA duration was 11.4 (8.7) years (n = 842), 68.7 % were RF positive (n = 719 with available data), and 64.5 % were anti-CCP positive (n = 456 with available data). Previous and concomitant treatments for the overall analysis population are shown in Table 2. Patients received a mean (SD) of 2.9 (1.5) prior cDMARDs, with 283/865 (32.7 %) patients having received more than three prior cDMARDs. An anti-TNF was the most frequent type of last biologic agent received prior to abatacept initiation (717/851 patients [84.3 %]) and 429/865 (49.6 %) patients had received at least two prior anti-TNF agents. The mean (SD) cumulative duration of the last biologic agent was 18.9 (22.2) months (n = 801). Secondary inefficacy (loss of efficacy after initial response) was the most common reason for discontinuation of the last biologic (400/847 [47.2 %] patients). Abatacept was most frequently initiated in combination with MTX (with or without other cDMARDs; 483/865 [55.8 %] patients). In patients who started or continued treatment with corticosteroids at abatacept initiation, median (SD) corticosteroid dose was 8.73 (11.62) mg/day (n = 645).

**Table 1:** Baseline demographics, disease characteristics and comorbidities (analysis population)

Characteristic	N = 865
Demographics	
Age	N = 865
Mean (SD), years	56.5 (12.1)
* <65 years, n (%)*	615 (71.1)
≥65 years, n (%)	250 (28.9)
Body mass index^a^	N = 818
Mean (SD), kg/m^2^	27.5 (5.8)
*<25 kg/m* ^*2*^ *, n (%)*	310 (37.9)
25– < 30 kg/m^2^, n (%)	275 (33.6)
30– < 35 kg/m^2^, n (%)	154 (18.8)
≥35 kg/m^2^, n (%)	79 (9.7)
Sex	N = 865
* Men, n (%)*	146 (16.9)
Women, n (%)	719 (83.1)
Country	N = 865
Canada, n (%)	163 (18.8)
*Germany, n (%)*	370 (42.8)
Greece, n (%)	110 (12.7)
Italy, n (%)	222 (25.7)
Disease characteristics	
RA duration	N = 842
Mean (SD), years	11.4 (8.7)
* ≤2 years, n (%)*	83 (9.9)
3–5 years, n (%)	169 (20.1)
6–10 years, n (%)	224 (26.6)
>10 years, n (%)	366 (43.5)
Tender joint count/28	N = 848
Mean (SD)	11.4 (7.3)
Swollen joint count/28	N = 858
Mean (SD)	7.9 (5.9)
HAQ-DI	N = 796
* <1.50, n (%)*	332 (41.7)
≥1.50, n (%)	464 (58.3)
DAS28 (ESR, otherwise CRP)	N = 793
*Remission or LDAS (≤3.2), n (%)*	24 (3.0)
MDAS (>3.2–5.1), n (%)	203 (25.6)
HDAS (>5.1), n (%)	440 (55.5)
Not done, n (%)	126 (15.9)
CDAI (calculated)	N = 865
*Remission, LDAS, or MDAS (≤22), n (%)*	196 (22.7)
HDAS (>22), n (%)	568 (65.7)
Missing, n (%)	101 (11.7)
Radiographic erosion (presence)	N = 750
*No, n (%)*	218 (29.1)
Yes, n (%)	532 (70.9)
CRP	N = 865
*<4 mg/L, n (%)*	224 (25.9)
4– < 10 mg/L, n (%)	172 (19.9)
10– < 26 mg/L, n (%)	204 (23.6)
≥26 mg/L, n (%)	182 (21.0)
Not done, n (%)	83 (9.6)
RF status	N = 852
* Negative, n (%)*	225 (26.4)
Positive, n (%)	494 (58.0)
Not available, n (%)	133 (15.6)
Anti-CCP status	N = 834
*Negative, n (%)*	162 (19.4)
Positive, n (%)	294 (35.3)
Not available, n (%)	378 (45.3)
Comorbidities	
Cardiovascular disorders	N = 865
*No, n (%)*	807 (93.3)
Yes, n (%)	58 (6.7)
Cardiac arrhythmia, n (%)	22 (2.5)
Cardiac valve disorder, n (%)	9 (1.0)
Coronary artery disorder, n (%)	23 (2.7)
Heart failure, n (%)	15 (1.7)
Myocardial disorder, n (%)	2 (0.2)
COPD	N = 865
*No, n (%)*	803 (92.8)
Yes, n (%)	62 (7.2)
Diabetes mellitus	N = 865
* No, n (%)*	753 (87.1)
Yes, n (%)	112 (12.9)
Tobacco use	N = 865
*No, n (%)*	757 (87.5)
Yes, n (%)	108 (12.5)
Infections and infestations	N = 865
*No, n (%)*	809 (93.5)
Yes, n (%)	56 (6.5)

Category in italics is the reference for univariate and multivariate analyses

The analysis population included patients treated in Canada, Germany, Greece, and Italy who had received at least one prior biologic agent and had a baseline clinical assessment no later than 8 days after the first administration of abatacept

*CCP* cyclic citrullinated peptide, *CDAI* Clinical Disease Activity Index, *CRP* C-reactive protein, *DAS* Disease Activity Score, *ESR* erythrocyte sedimentation rate, *HAQ-DI* Health Assessment Questionnaire-Disability Index, *HDAS* high Disease Activity Score, *LDAS* low Disease Activity Score, *MDAS* moderate Disease Activity Score, *RA* rheumatoid arthritis, *RF* rheumatoid factor, *SD* standard deviation

^a^World Health Organization body mass index classification: underweight/normal if <25 kg/m^2^, overweight if 25– < 30 kg/m^2^, obese class I if 30– < 35 kg/m^2^, and obese class II/III if ≥35 kg/m^2^ [21]

**Table 2:** Previous and concomitant medications (analysis population)

Treatment parameter	N = 865
Previous treatments	
Number of prior DMARDs	N = 865
*0–3, n (%)*	582 (67.3)
>3, n (%)	283 (32.7)
Number of prior anti-TNF agents	N = 865
* ≥2, n (%)*	429 (49.6)
<2, n (%)	436 (50.4)
Type of biologic agent	N = 851
*Other MOA, n (%)*	134 (15.8)
Anti-TNF agent	717 (84.3)
Reason for discontinuation of last biologic	N = 847
*Intolerance, n (%)*	190 (22.4)
Primary inefficacy, n (%)^a^	203 (24.0)
Secondary inefficacy, n (%)^†^	400 (47.2)
Major improvement + other reasons, n (%)	54 (6.4)
Concomitant therapies	
Abatacept treatment pattern at initiation	N = 865
* Monotherapy, n (%)*	201 (23.2)
Combination with MTX (± other DMARDs), n (%)	483 (55.8)
Combination with other DMARDs, n (%)	181 (20.9)
Corticosteroid treatment pattern at abatacept initiation (versus before initiation)^‡^	N = 865
*No corticosteroids, n (%)*	202 (25.4)
Continuous use of corticosteroids, n (%)	491 (56.8)
Stop corticosteroid use, n (%)	18 (2.1)
Introduction of corticosteroids, n (%)	154 (17.8)

Category in italics is the reference for univariate and multivariate analyses

The analysis population included patients treated in Canada, Germany, Greece, and Italy who had received at least one prior biologic agent and had a baseline clinical assessment no later than 8 days after the first administration of abatacept. Patient population includes 17 patients who did not receive prior treatment with an anti-TNF agent but a biologic agent with another MOA

*DMARD* disease-modifying anti-rheumatic drug, *MOA* mechanism of action, *MTX* methotrexate, *TNF* tumor necrosis factor

^a^Failure to respond; ^†^Loss of efficacy after initial response. ^‡^Mean (standard deviation) corticosteroid dose was 8.73 (11.62) mg/day in patients who continued use of corticosteroids or who started corticosteroids at abatacept initiation (n = 645)

Further analysis of baseline data revealed that most characteristics were broadly comparable across the countries assessed. However, some differences between countries were observed in terms of patient demographics, disease characteristics, and previous and concomitant therapies, as highlighted in the supplementary information (Additional file 2: Table S2).

In a *post hoc* analysis, socio-demographics, disease characteristics and comorbidities at abatacept initiation were compared in patients who were anti-CCP antibody seropositive versus seronegative. This analysis included all patients enrolled in ACTION between May 2008 and January 2011 who had received at least one prior biologic agent. Patients who were seropositive versus seronegative (n = 472 and n = 253, respectively) had lower mean (SD) body weight (74.0 [16.6] vs 77.2 [17.8] kg, *p* = 0.014) and body mass index (26.9 [5.3] vs 28.5 [6.0] kg/m^2^, *p* = 0.001), longer RA duration (12.1 [8.9] vs 10.9 [9.5] years, *p* = 0.018) and more severe disease (erythrocyte sedimentation rate: 37.5 [24.1] vs 30.1 [23.8] mm/h, *p* < 0.001; radiographic erosion: 74.9 % vs 59.5 %, *p* < 0.001). Concomitant treatments at abatacept initiation were similar for patients who were seropositive versus seronegative except for a numerically higher percentage of patients with anti-CCP seropositivity who received concomitant corticosteroids (77.3 % vs 71.1 %, *p* = 0.072).

### Retention rate

**Fig. 1:**
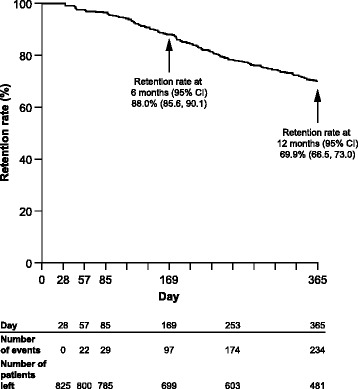
Retention rate over 12 months of abatacept treatment (analysis population). The retention rate estimate and 95 % CIs were computed using the Kaplan–Meier method. An event was defined as a discontinuation reported by the physician at any follow-up visit; patients who did not reach the considered time point were censored at the date of last data available; patients with only baseline data were considered as censored at first infusion date. The analysis population included patients treated in Canada, Germany, Greece, and Italy who had received at least one prior biologic agent and had a baseline clinical assessment no later than 8 days after the first administration of abatacept. CI, confidence interval

The overall retention rate over 12 months is shown in Fig. 1. The retention rate (95 % CI) was 88.0 % (85.6, 90.1) at 6 months and 69.9 % (66.5, 73.0) at 12 months. The overall retention rates (95 % CI) per country at 6 and 12 months, respectively, were 96.8 % (90.4, 99.0) and 87.7 % (78.1, 93.2) in Greece, 90.4 % (85.5, 93.7) and 80.3 % (74.1, 85.1) in Italy, 89.2 % (83.3, 93.2) and 64.6 % (56.4, 71.8) in Canada, and 83.6 % (79.3, 87.1) and 61.3 % (55.9, 66.3) in Germany.

Over 12 months, 21.2 % of patients discontinued abatacept because of inefficacy (EULAR response) and 6.8 % discontinued because of intolerance.

### Univariate analysis

**Fig. 2:**
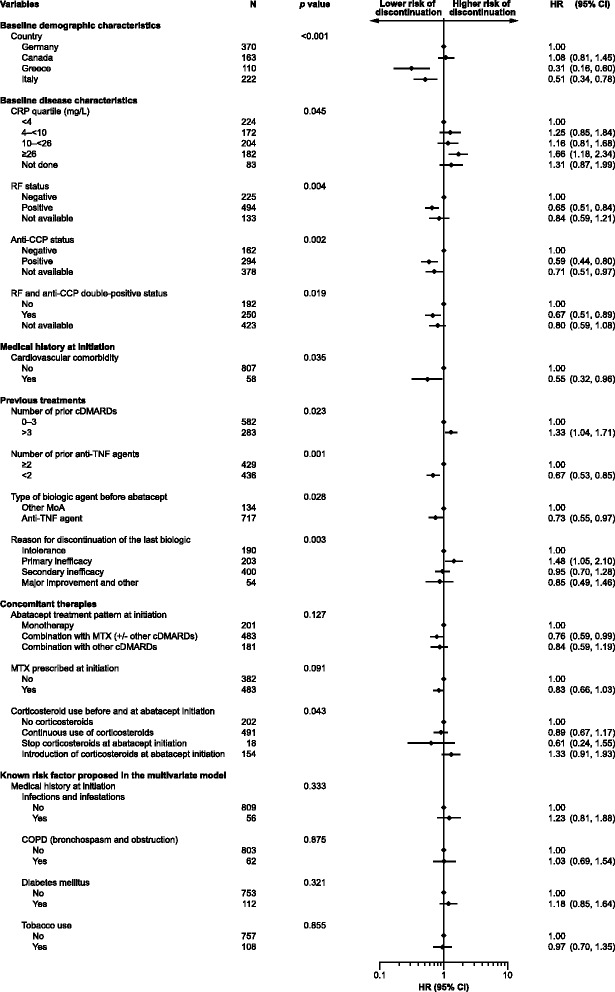
Univariate model of abatacept discontinuation. Results are presented for variables retained in the model at the 20 % level. HRs are presented with corresponding 95 % CIs. An HR >1 indicates an increased risk of abatacept discontinuation. An HR <1 indicates a decreased risk of abatacept discontinuation. HRs are significant when the 95 % CIs do not overlap 1. The patient population included 17 patients who were anti-TNF naïve and who had previously received treatment with a biologic with a different mechanism of action. CCP, cyclic citrullinated peptide; cDMARD, conventional disease-modifying anti-rheumatic drug; CI, confidence interval; COPD, chronic obstructive pulmonary disease; CRP, C-reactive protein; HR, hazard ratio; MTX, methotrexate; RF, rheumatoid factor; TNF, tumor necrosis factor

Known risk factors of COPD, diabetes mellitus, tobacco use, and infection or infestation were proposed in the model even though they were not significant. Based on univariate analyses, 13 variables were eligible to enter the multivariate model (*p* ≤ 0.20; Fig. 2). Among them, two variables (RF and anti-CCP double positivity, and concomitant MTX) were not entered into the multivariate model due to collinearity with other prognostic variables.

### Multivariate analysis

**Fig. 3:**
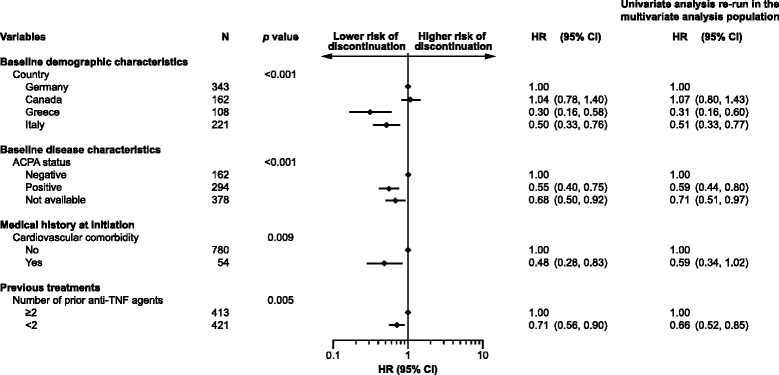
Multivariate model of abatacept discontinuation (n = 834). Results are presented for variables retained in the model at the 10 % level. HRs are presented with corresponding 95 % CIs. An HR >1 indicates an increased risk of abatacept discontinuation. An HR <1 indicates a decreased risk of abatacept discontinuation. HRs are significant when the 95 % CIs do not overlap 1. The patient population included 17 patients who were anti-TNF naïve and had previously received treatment with a biologic with a different mechanism of action. ACPA, anti-citrullinated protein antibody; CI, confidence interval; HR, hazard ratio; TNF, tumor necrosis factor

Overall, 834/865 (96.4 %) patients were considered in the multivariate model (Fig. 3). Patients had a significantly lower risk of abatacept discontinuation if they were anti-CCP positive (HR [95 % CI]: 0.55 [0.40, 0.75], *p* < 0.001), had failed <2 anti-TNF agents (0.71 [0.56, 0.90] versus ≥2 anti-TNF agents, *p* = 0.005), or had a cardiovascular comorbidity at abatacept initiation (0.48 [0.28, 0.83], *p* = 0.009). Patients in Greece and Italy were less likely to discontinue abatacept than patients in Germany and Canada (HR [95 % CI] versus Germany: 0.30 [0.16, 0.58] for Greece, 0.50 [0.33, 0.76] for Italy, 1.04 [0.78, 1.40] for Canada, *p* < 0.001). No significant interaction was found across prognostic factors. CRP level at baseline, RF status at baseline, and type of previous anti-TNF failure were significant in the univariate analysis (*p* < 0.05) but were no longer significant in the multivariate analysis. Abatacept treatment pattern at initiation (monotherapy, combination with MTX or combination with other cDMARDs) was entered into the multivariate model but was not found to be significant. No effect was observed in either univariate or multivariate analyses for infection at initiation or disease duration. The sensitivity analysis, including all variables that were significant in the univariate analysis with no variable selection in the multivariate step, was consistent.

## Discussion

This is the first international, prospective study to evaluate the prognostic factors for abatacept retention in a real-world setting in patients with RA who had received at least one prior biologic agent. In this interim analysis, the 6- and 12-month abatacept retention rates were 88.0 % and 69.9 %, respectively. Abatacept retention rates in ACTION were numerically within the range reported in other published studies [11, 13, 22]. The 12-month retention rates for abatacept were within the range of those reported for tocilizumab [22–25] and for anti-TNFs [26].

Here, we report predictive factors for abatacept retention at 12 months identified from a cohort of patients from the ACTION study who had received at least one prior biologic agent: anti-CCP positivity, failing <2 prior anti-TNF agents, and cardiovascular comorbidity at initiation were associated with higher retention. Differences in retention between some countries were also observed. The percentage of patients receiving abatacept as monotherapy in this study (23.2 %) was broadly consistent with that observed in biologic registries (approximately 30 %) [27].

In this study, anti-CCP positivity was associated with improved abatacept retention, compared with anti-CCP negative status. In addition, RF seropositivity was significant in the univariate analysis but not in the multivariate analysis. Anti-CCP positivity has previously been shown to be a predictor of clinical response to abatacept in patients with RA [11, 28]. The mechanisms underlying this association remain to be further elucidated, although some initial findings suggest that the very early effect of abatacept on T-cell modulation is an important mechanism of action in patients with anti-CCP positivity [29]. In the present study, patients with exposure to at least two prior anti-TNFs were more likely to discontinue abatacept than patients with exposure <2 prior anti-TNFs. Patients with prior exposure to multiple anti-TNFs are likely to have advanced RA and may find it difficult to benefit from any therapy; furthermore, previous studies have shown that no or low prior exposure to biologic agents is associated with longer drug survival in patients receiving tocilizumab or anti-TNFs [14, 23, 30]. The reason for discontinuation of the last biologic agent prior to abatacept initiation was a significant predictor of abatacept retention in the univariate but not the multivariate analysis. In a subgroup analysis of the ATTAIN (Abatacept Trial in Treatment of Anti-TNF INadequate responders) trial, the reason for prior anti-TNF failure was not associated with differences in the efficacy of abatacept over 6 months [31].

Cardiovascular comorbidity at initiation (including cardiac arrhythmia, cardiac valve disorders, coronary artery disorders, heart failure, and myocardial disorders) was associated with a decreased risk of abatacept discontinuation compared with not having cardiovascular comorbidity. The long-term safety profile of abatacept is well established in RA [32]. Abatacept does not have any special warnings or contraindications in patients with cardiovascular diseases. In contrast, adalimumab, certolizumab pegol, golimumab and infliximab are contraindicated in patients with moderate-to-severe heart failure (New York Heart Association class III/IV) [33–36] and etanercept has a special warning in patients with congestive heart failure [37]; this may result in limited options for switching treatment in patients with a history of cardiovascular comorbidity. Channeling may have been introduced at abatacept initiation and comorbidities may have been underreported.

Differences in retention were observed between countries in the multivariate analysis. Patients in Greece and Italy were less likely to discontinue abatacept than patients in Germany and Canada; these differences in abatacept retention may represent differences in patient populations and disease characteristics, access to biologic agents, and national guidelines for the management of RA. Although covering other European countries, country was found to impact abatacept retention in a pooled analysis of 9 European registries [38, 39]. Baseline demographics and disease characteristics were broadly comparable across the countries included in this analysis, although patients in Canada had higher swollen joint counts and were less likely to exhibit radiographic erosion than patients in Germany, Italy, and Greece. Patients in Italy were less likely to have received ≥2 prior biologic agents compared with patients in the other countries assessed, and rates of concomitant corticosteroid use differed markedly between countries (48.5 % in Canada compared with 69.1 %, 78.8 %, and 85.1 % in Greece, Italy, and Germany, respectively). In addition, several studies have reported a lower incidence and prevalence of RA, as well as disease activity, in southern European Union (EU) countries versus northern EU countries [40, 41]. Access to biologic agents may be related to gross domestic product per capita and healthcare system structure, including the number of rheumatologists per inhabitant [38, 42, 43]. Treatment guidance for the management of RA is available in most countries and may have been developed taking into account gross domestic product and disease parameters (e.g. disease activity, poor prognostic factors) [44]. Finally, patient management is also dependent on the type of practice (evidence-based versus routine practice). Together, these elements may partly explain differences in retention between countries, independently of patient characteristics. However, such detailed information was not specifically collected and is probably aggregated in the variable ‘country’, including influence of genetic background and environmental factors.

The limitations of this study are similar to other studies using uncontrolled, real-world data. There was no active comparator in this study and there are few examples of real-world data comparing abatacept with other biologic agents, or comparing other biologic agents. Owing to the real-world design of ACTION, a selection bias based on disease severity or adverse events is plausible. To minimize patient selection bias, participating physicians enrolled subsequent patients who were eligible as per the inclusion and exclusion criteria. To ensure that ACTION did not interfere with a physician’s routine clinical practice, the decision to treat a patient with abatacept was made prior to enrollment in the study. A process of random selection of study investigators from a comprehensive list of rheumatologists was used to obtain a geographically balanced group of investigators in each country who were representative of rheumatologists who treat patients with IV biologics.

This analysis of patients from the ACTION study included a large number of patients in an international real-life setting (n = 865), which permitted inclusion of a comprehensive list of all relevant prognostic factors. Most previous studies of abatacept in clinical practice have explored only national registries from single countries [11, 22, 45]. As this was an interim analysis, further analyses are warranted to confirm these findings.

Although many studies have identified predictive factors of clinical response to biologic agents, few have been confirmed, in particular for abatacept [10]. Even fewer studies have identified prognostic factors of abatacept retention. Similar trends were found in ACTION and in the pooled analysis of 9 European registries [39]. Given the range of biologic agents now available for the treatment of RA, the identification of patients who will benefit from a specific therapy is of interest and may aid realistic cost-effectiveness estimates. Therefore, the identification of prognostic factors of clinical response and retention are of growing importance, as highlighted by the 2013 EULAR recommendations [8].

## Conclusions

Abatacept retention rates at 6 and 12 months were high and were consistent with previous studies in national registries. Anti-CCP positivity and less than two prior anti-TNFs were predictors of higher abatacept retention. There are no contraindications or special warnings for abatacept in patients with cardiovascular comorbidities based on study results and registries, and the results of this study suggest that abatacept may be a good treatment option in these patients. Abatacept retention varied between countries and may reflect differences in healthcare systems. The prognostic factors described in this study are derived from an international cohort of patients and may support therapy decisions made by physicians when considering abatacept for the treatment of moderate-to-severe RA. Further analyses on the complete 2-year dataset are expected to confirm these findings. Overall, these findings indicate that abatacept may be a treatment of choice in patients at high risk of disease progression.

## Additional files

Additional file 1:
**Table S1.** List of ethics committee approvals for the ACTION study in countries that enrolled patients between May 2008 and January 2011. (DOCX 28 kb) Additional file 2:
**Table S2.** Baseline demographics, disease characteristics, comorbidities, and previous and concomitant medications by country (analysis population). Baseline demographics, disease characteristics, comorbidities, and previous and concomitant medications by country in the analysis population. (DOCX 39 kb)

## Abbreviations

ACTION: AbataCepT In rOutiNe clinical practice
ATTAIN: Abatacept Trial in Treatment of Anti-TNF INadequate responders
CCP: Cyclic citrullinated peptide
cDMARD: conventional disease-modifying anti-rheumatic drug
CI: Confidence interval
COPD: Chronic obstructive pulmonary disease
CRP: C-reactive protein
DAS: Disease Activity Score
DMARD: Disease-modifying anti-rheumatic drug
EU: European Union
EULAR: European League Against Rheumatism
HR: Hazard ratio
IV: Intravenous
MTX: Methotrexate
ORA: Orencia and Rheumatoid Arthritis
RA: Rheumatoid arthritis
RF: Rheumatoid factor
SD: Standard deviation
SOC: System Organ Class
TNF: Tumor necrosis factor
