# Supplementary material for: Prognostic factors for abatacept retention in patients who received at least one prior biologic agent: an interim analysis from the observational, prospective ACTION study
Source: BMC Musculoskelet Disord. 2015 Jul 30;16:176. doi: 10.1186/s12891-015-0636-9 (PMC4521342; doi:10.1186/s12891-015-0636-9)
Supplement: Additional file 1: — Table S1. List of ethics committee approvals for the ACTION study in countries that enrolled patients between May 2008 and January 2011. (DOCX 28 kb) [file 12891_2015_636_MOESM1_ESM.docx]

**Table S1 List of ethics committee approvals for the ACTION study in countries that enrolled patients between May 2008 and January 2011**

| **Country** | **Type of ethics committee** | **Name** |
| --- | --- | --- |
| Austria | Not applicable* |  |
| Belgium | Local ethics committee | AZ Groeningen Ethisch Comité |
| Belgium | Local ethics committee | Comité d’Ethique du Grand Hôpital de Charleroi |
| Belgium | Local ethics committee | Comité d’Ethique CHU de Liège |
| Belgium | Local ethics committee | Ethische Commissie n° OG-057, AZ Damiaan Oostende |
| Belgium | Local ethics committee | Comité d’Ethique Centre Hospitalier Peltzer – La Tourelle |
| Belgium | Local ethics committee | Aalst OG 052 |
| Belgium | Local ethics committee | Ethics Committee AZ Sint-Lucas vzw |
| Belgium | Local ethics committee | Commissie Medische Ethiek Universitaire Ziekenhuizen K.U.Leuven |
| Canada | Central Ethics (Central Institutional Review Board) | ON-IRB/REB (Toronto, Ontario) |
| Canada | College of Physicians and Surgeons of Alberta (CPSA) | College of Physicians and Surgeons of Alberta (CPSA) |
| Canada | Institutional Review Board | Chus-Hopital de Fleurimont, Dept of Rheumatology |
| Canada | Local ethics committee | Mount Sinai Hospital'Joseph and Wolf Lebovic Building |
| Canada | Local ethics committee | Chus-Hopital de Fleurimont, Dept of Medicine |
| Canada | Local ethics committee | Royal University Hospital, Dept of Rheumatology |
| Canada | Local ethics committee | St Clare’s Mercy Hospital, Dept of Rheumatology |
| Canada | Local ethics committee | Hopital Maisonneuve - Rosemont |
| Czech Republic | Not applicable* |  |
| Denmark | Not applicable* |  |
| Germany | Central ethics committee | Ethik-Kommission  der Bayerischen Landesärztekammer  Mühlbaurstr.16  D-81677 München |
| Greece | Local scientific council | General Hospital of Athens "Laiko" |
| Greece | Local scientific council | University General Hospital of Heraklion |
| Greece | Local scientific council | ACHEPA Hospital of Thessaloniki |
| Greece | Local scientific council | University Hospital of Larissa |
| Greece | Local scientific council | General Hospital of Thessaloniki, "G. Papanikolaou" |
| Greece | Local scientific council | General Hospital of Athens "Ippokratio" |
| Greece | Local scientific council | University Hospital of Patra |
| Greece | Local scientific council | "Evagelismos" General Hospital |
| Greece | Local scientific council | "NIMTS" Hospital |
| Greece | Local scientific council | 251 Hellenic Airforce General Hospital |
| Greece | Local scientific council | General Hospital of Voula "Asklipeion" |
| Greece | Local scientific council | Naval Hospital |
| Greece | Local scientific council | St. Paul's Hospital |
| Greece | Local scientific council | "Euromedica" Clinic |
| Greece | Local scientific council | General Hospital of Patras "Agios Andreas" |
| Greece | Local scientific council | General Hospital of Krditsa |
| Greece | Local scientific council | KAT Hospital |
| Greece | Local scientific council | Diavalkaniko Medical Center |
| Greece | Local scientific council | General Hospital of Kavalas |
| Greece | Local scientific council | Scientific council of Euromedica-Central Clinic of Thessaloniki |
| Greece | Local scientific council | Scientific Council of the General Hospital of Patras “Agios Andreas” |
| Greece | Local scientific council | Scientific Council of the University General Hospital of Larisa |
| The Netherlands | Not applicable* |  |
| Italy | Local ethics committee | Comitato Etico Locale per la Sperimentazione Clinica dei Medicinalic/o U.O.C. Farmacia A.O.U. Senese |
| Italy | Local ethics committee | Comitato Etico della ASL 3 Genovese |
| Italy | Local ethics committee | Comitato di Bioetica Fondaz. IRCCS Pol. San Matteo di Pavia |
| Italy | Local ethics committee | Comitato Etico A.O.U. Pol. Vittorio Emanuele – Catania |
| Italy | Local ethics committee | Comitato di Bioetica della Prov. Religiosa S. Pietro-Fatebenefratelli di Roma |
| Italy | Local ethics committee | Comitato Etico dell'A.O.U. "San Martino" di Genova |
| Italy | Local ethics committee | Comitato di Bioetica per la Sperimentazione Clinica dei Medicinali A.O.U. Pisana |
| Italy | Local ethics committee | Comitato Etico A.O. "Bianchi-Melacrino-Morelli" - Reggio Calabria |
| Italy | Local ethics committee | Comitato Etico Scientifico A.O.U. Pol. G. Martino di Messina |
| Italy | Local ethics committee | Comitato di Etica dell'A.O. Ospedale Sant'Anna di Como |
| Italy | Local ethics committee | Comitato Etico Locale della A.O.U. Careggi di Firenze |
| Italy | Local ethics committee | Comitato Etico dell'Azienda Sanitaria Unica delle Marche - Ancona |
| Italy | Local ethics committee | Comitato Etico dell'A.O. S. Andrea di Roma |
| Italy | Local ethics committee | Comitato Etico dell'A.O. Ospedale Civile di Legnano |
| Italy | Local ethics committee | Comitato Etico Aziendale A.O.U. S. Maria della Misericordia - Udine |
| Italy | Local ethics committee | Comitato Etico Unico per la Prov. di Parma |
| Italy | Local ethics committee | Comitato Etico Indipendente A.O.U. Pol. Tor Vergata - Roma |
| Italy | Local ethics committee | Comitato Etico Indip. Locale A.O. "Ospedale Policlinico Consorziale" di Bari |
| Italy | Local ethics committee | Comitato Etico A.O. Cannizzaro - Catania |
| Italy | Local ethics committee | Comitato Etico Indipendente A.O.U. Ospedali Riuniti di Foggia |
| Italy | Local ethics committee | Comitato Etico dell'Università Cattolica, Pol. Agostino Gemelli - Roma |
| Italy | Local ethics committee | Comitato Etico Segreteria Tecnico Scientifica c/o Serv. di Farmacia - Ospedale San Camillo - Roma |
| Italy | Local ethics committee | Comitato Etico ASL di Lecce |
| Italy | Local ethics committee | Comitato Etico dell'A.O. Regionale "San Carlo" di Potenza |
| Italy | Local ethics committee | Comitato Etico dell'A.O. Villa Sofia - CTO di Palermo |
| Italy | Local ethics committee | Comitato Etico per la Sperimentazione Clinica dei Farmaci AUSL di Pescara |
| Italy | Local ethics committee | Comitato Etico-Scientifico A.O. Ospedale Niguarda Cà Granda - Milano |
| Italy | Local ethics committee | Comitato Etico dell'A.O.U. Pol. di Cagliari |
| Italy | Local ethics committee | Comitato Etico Interaziendale A.O.U. San Giovanni Battista di Torino - A.O. "C.T.O. Maria Adelaide di Torino" |
| Italy | Local ethics committee | Comitato Etico Indipendente A.O. di Rilievo Nazionale Gaetano Rummo - Benevento |
| Italy | Local ethics committee | Comitato Etico ASL 1 c/o P.O. S. Salvatore - L'Aquila |
| Italy | Local ethics committee | Comitato Etico Aziende Sanitarie (C.E.A.S.) Umbria - Ellera di Corciano (PG) |
| Italy | Local ethics committee | Comitato Etico per la Sperimentazione Clinica dei Medicinali dell'A.O.U. Integrata di Verona |
| Italy | Local ethics committee | Comitato Etico dell'A.O.U. Seconda Università degli Studi di Napoli |
| Italy | Local ethics committee | Comitato Etico Provinciale di Modena |
| Italy | Local ethics committee | Comitato Etico A.O. Istituto Ortopedico Gaetano Pini - Milano |
| Italy | Local ethics committee | Comitato di Bioetica Azienda Ospedaliera di Rilievo Nazionale e di Alta Specializzazione Civico e Benfratelli - Giovanni Di Cristina - Maurizio Ascoli - Palermo |
| Italy | Local ethics committee | Comitato Etico Provinciale A.O.U. di Ferrara |
| Italy | Local ethics committee | Comitato Etico dell'Azienda Sanitaria Provinciale di Cosenza |
| Italy | Local ethics committee | Comitato Etico A.O. Spedali Civili di Brescia |
| Italy | Local ethics committee | Comitato di Etica della ASL SA/1 di Nocera Inferiore |
| Italy | Local ethics committee | Comitato Etico ASL NA1 CENTROCentro Direz. Isola F9 - Napoli |
| Italy | Local ethics committee | Comitato Etico Interaziendale dell'A.O. OIRM/S. Anna di Torino |
| Italy | Local ethics committee | Comitato Etico Indip. dell'A.O.U. Pol. S. Orsola-Malpighi - Bologna |
| Italy | Local ethics committee | Comitato Etico A.O. Ospedale Luigi Sacco - Milano |

*Full submission to an ethics committee was not required in these countries owing to the non-interventional design of the study.
