# Supplementary material for: Prognostic factors for abatacept retention in patients who received at least one prior biologic agent: an interim analysis from the observational, prospective ACTION study
Source: BMC Musculoskelet Disord. 2015 Jul 30;16:176. doi: 10.1186/s12891-015-0636-9 (PMC4521342; doi:10.1186/s12891-015-0636-9)
Supplement: Additional file 2: — Table S2. Baseline demographics, disease characteristics, comorbidities, and previous and concomitant medications by country (analysis population). Baseline demographics, disease characteristics, comorbidities, and previous and concomitant medications by country in the analysis population. (DOCX 39 kb) [file 12891_2015_636_MOESM2_ESM.docx]

Table S2 **Baseline demographics, disease characteristics, comorbidities, and previous and concomitant medications by country (analysis population)**

| **Characteristic** | **Canada** | **Greece** | **Germany** | **Italy** |
| --- | --- | --- | --- | --- |
| **Demographics** | | | | |
| **Age** Mean (SD), years *<65 years, n (%)* ≥65 years, n (%) | N = 163 57.3 (11.7) 114 (69.9) 49 (30.1) | N = 110 58.9 (11.9) 68 (61.8) 42 (38.2) | N = 370 55.4 (12.6) 270 (73.0) 100 (27.0) | N = 222 56.7 (11.3) 163 (73.4) 59 (26.6) |
| **Body mass index*** Mean (SD), kg/m^2^ *<25 kg/m^2^, n (%)* 25‒<30 kg/m^2^, n (%) 30‒<35 kg/m^2^, n (%) ≥35 kg/m^2^, n (%) | N = 134 29.2 (7.0) 40 (29.9) 39 (29.1) 33 (24.6) 22 (16.4) | N = 102 29.1 (4.9) 17 (16.7) 48 (47.1) 21 (20.6) 16 (15.7) | N = 360 27.3 (5.7) 141 (39.2) 126 (35.0) 64 (17.8) 29 (8.1) | N = 222 26.2 (5.0) 112 (50.5) 62 (27.9) 36 (16.2) 12 (5.4) |
| **Sex** *Men, n (%)* Women, n (%) | N = 163 24 (14.7) 139 (85.3) | N = 110 22 (20.0) 88 (80.0) | N = 370 72 (19.5) 298 (80.5) | N = 222 28 (12.6) 194 (87.4) |
| **Disease characteristics** | | | | |
| **RA duration** Mean (SD), years *≤2 years, n (%)* 3‒5 years, n (%) 6‒10 years, n (%) >10 years, n (%) | N = 162 10.7 (9.1) 15 (9.3) 39 (24.1) 46 (28.4) 62 (38.3) | N = 106 10.4 (8.4) 17 (16.0) 22 (20.8) 27 (25.5) 40 (37.7) | N = 358 12.0 (8.9) 30 (8.4) 69 (19.3) 87 (24.3) 172 (48.0) | N = 216 11.4 (8.0) 21 (9.7) 39 (18.1) 64 (29.6) 92 (42.6) |
| **Tender joint count/28** Mean (SD) | N = 150 14.5 (7.4) | N = 110 12.3 (7.0) | N = 366 10.2 (7.4) | N = 222 10.9 (6.7) |
| **Swollen joint count/28** Mean (SD) | N = 159 10.8 (6.7) | N = 110 6.9 (5.1) | N = 367 7.6 (5.9) | N = 222 6.9 (5.0) |
| **HAQ-DI** *<1.50, n (%)* ≥1.50, n (%) | N = 150 48 (32.0) 102 (68.0) | N = 91 40 (44.0) 51 (56.0) | N = 334 155 (46.4) 179 (53.6) | N = 221 89 (40.3) 132 (59.7) |
| **DAS28 (ESR, otherwise CRP)** *Remission or LDAS (<3.2), n (%)* MDAS (3.2‒5.1), n (%) HDAS (>5.1), n (%) Not done, n (%) | N = 161 0 9 (5.6) 42 (26.1) 110 (68.3) | N = 107 2 (1.9) 19 (17.8) 70 (65.4) 16 (15.0) | N = 303 18 (5.9) 104 (34.3) 181 (59.7) 0 | N = 222 4 (1.8) 71 (32.0) 147 (66.2) 0 |
| **CDAI (calculated)** *Remission, LDAS, or MDAS (≤22), n (%)* HDAS (>22), n (%) Missing, n (%) | N = 163  14 (8.6) 70 (42.9) 79 (48.5) | N = 110  16 (14.5) 82 (74.5) 12 (10.9) | N = 370  104 (28.1) 257 (69.5) 9 (2.4) | N = 222  62 (27.9) 159 (71.6) 1 (0.5) |
| **Radiographic erosion (presence)** *No, n (%)* Yes, n (%) | N = 147 72 (49.0) 75 (51.0) | N = 108 41 (38.0) 67 (62.0) | N = 273 59 (21.6) 214 (78.4) | N = 222 46 (20.7) 176 (79.3) |
| **CRP** *<4 mg/L, n (%)* 4‒<10 mg/L, n (%) 10‒<26 mg/L, n (%) ≥26 mg/L, n (%) Not done, n (%) | N = 163 32 (19.6) 33 (20.2) 28 (17.2) 36 (22.1) 34 (20.9) | N = 110 33 (30.0) 23 (20.9) 21 (19.1) 23 (20.9) 10 (9.1) | N = 370 77 (20.8) 72 (19.5) 102 (27.6) 93 (25.1) 26 (7.0) | N = 222 82 (36.9) 44 (19.8) 53 (23.9) 30 (13.5) 13 (5.9) |
| **RF status** *Negative, n (%)* Positive, n (%) Not available, n (%) | N = 161 49 (30.4) 82 (50.9) 30 (18.6) | N = 109 31 (28.4) 64 (58.7) 14 (12.8) | N = 361 82 (22.7) 215 (59.6) 64 (17.7) | N = 221 63 (28.5) 133 (60.2) 25 (11.3) |
| **Anti-CCP status** *Negative, n (%)* Positive, n (%) Not available, n (%) | N = 162 27 (16.7) 25 (15.4) 110 (67.9) | N = 108 20 (18.5) 29 (26.9) 59 (54.6) | N = 343 68 (19.8) 150 (43.7) 125 (36.4) | N = 221 47 (21.3) 90 (40.7) 84 (38.0) |
| **Comorbidities** | | | | |
| **Cardiovascular disorders** *No, n (%)* Yes, n (%) Cardiac arrhythmias, n (%) Cardiac valve disorders, n (%) Coronary artery disorders, n (%) Heart failures, n (%) Myocardial disorders, n (%) | N = 163 149 (91.4) 14 (8.6) 5 (3.1) 2 (1.2) 6 (3.7) 4 (2.5) 0 | N = 110 102 (92.7) 8 (7.3) 3 (2.7) 0 3 (2.7) 3 (2.7) 0 | N = 370 338 (91.4) 32 (8.6) 11 (3.0) 6 (1.6) 14 (3.8) 8 (2.2) 2 (0.5) | N = 222 218 (98.2) 4 (1.8) 3 (1.4) 1 (0.5) 0 0 0 |
| **COPD** *No, n (%)* Yes, n (%) | N = 163 154 (94.5) 9 (5.5) | N = 110 103 (93.6) 7 (6.4) | N = 370 336 (90.8) 34 (9.2) | N = 222 210 (94.6) 12 (5.4) |
| **Diabetes mellitus** *No, n (%)* Yes, n (%) | N = 163 141 (86.5) 22 (13.5) | N = 110 93 (84.5) 17 (15.5) | N = 370 317 (85.7) 53 (14.3) | N = 222 202 (91.0) 20 (9.0) |
| **Tobacco use** *No, n (%)* Yes, n (%) | N = 163 139 (85.3) 24 (14.7) | N = 110 97 (88.2) 13 (11.8) | N = 370 330 (89.2) 40 (10.8) | N = 222 191 (86.0) 31 (14.0) |
| **Infections and infestations** *No, n (%)* Yes, n (%) | N = 163 152 (93.3) 11 (6.7) | N = 110 108 (98.2) 2 (1.8) | N = 370 333 (90.0) 37 (10.0) | N = 222 216 (97.3) 6 (2.7) |
| **Previous treatments** | | | | |
| **Number of prior DMARDs** *0–3, n (%)* >3, n (%) | N = 163 96 (58.9) 67 (41.1) | N = 110 98 (89.1) 12 (10.9) | N = 370 229 (61.9) 141 (38.1) | N = 222 159 (71.6) 63 (28.4) |
| **Number of prior anti-TNF agents** *<2, n (%)* ≥2, n (%) | N = 163 83 (50.9) 80 (49.1) | N = 110 59 (53.6) 51 (46.4) | N = 370 159 (43.0) 211 (57.0) | N = 222 135 (60.8) 87 (39.2) |
| **Type of biologic agent** *Other MOA, n (%)* Anti-TNF agent | N = 163 19 (11.7) 144 (88.3) | N = 109 16 (14.7) 93 (85.3) | N = 357 77 (21.6) 280 (78.4) | N = 222 22 (9.9) 200 (90.1) |
| **Reason for discontinuation of last biologic** *Intolerance, n (%)* Primary inefficacy, n (%)^†^ Secondary inefficacy, n (%)^‡^ Major improvement + other reasons, n (%) | N = 162 38 (23.5) 37 (22.8) 75 (46.3) 12 (7.4) | N = 107 21 (19.6) 24 (22.4) 60 (56.1) 2 (1.9) | N = 356 81 (22.8) 102 (28.7) 145 (40.7) 28 (7.9) | N = 222 50 (22.5) 40 (18.0) 120 (54.1)  12 (5.4) |
| **Concomitant therapies** | | | | |
| **Abatacept treatment pattern at initiation** *Monotherapy, n (%)* Combination with MTX (± DMARDs), n (%) Combination with other DMARDs, n (%) | N=163 39 (23.9)  94 (57.7)  30 (18.4) | N=110 8 (7.3)  65 (59.1)  37 (33.6) | N=370 100 (27.0)  187 (50.5)  83 (22.4) | N=222 54 (24.3)  137 (61.7)  31 (14.0) |
| **Corticosteroid treatment pattern at abatacept initiation (versus before initiation)** *No corticosteroids, n (%)* Continuous use of corticosteroids, n (%) Stop corticosteroid use, n (%) Introduction of corticosteroids, n (%) | N=163 79 (48.5)  65 (39.9) 5 (3.1) 14 (8.6) | N=110 29 (26.4)  63 (57.3) 5 (4.5) 13 (11.8) | N=370 53 (14.3)  204 (55.1) 2 (0.5) 111 (30.0) | N=222 41 (18.5)  159 (71.6) 6 (2.7) 16 (7.2) |

*World Health Organization body mass index classification: underweight/normal if <25 kg/m^2^, overweight if 25–<30 kg/m^2^, obese class I if 30–<35 kg/m^2^, and obese class II/III if ≥35 kg/m^2^ [1].^†^Failure to respond. ^‡^Loss of efficacy after initial response.

Category in italics is the reference for univariate and multivariate analyses.

The analysis population included patients treated in Canada, Germany, Greece, and Italy who had received at least one prior biologic agent and had a baseline clinical assessment no later than 8 days after the first administration of abatacept.

CCP, cyclic citrullinated peptide; CDAI, Clinical Disease Activity Index; COPD, chronic obstructive pulmonary disease; CRP, C-reactive protein; DAS, Disease Activity Score; DMARD, disease-modifying antirheumatic drug; ESR, erythrocyte sedimentation rate; HAQ-DI, Health Assessment Questionnaire-Disability Index; HDAS, high Disease Activity Score; LDAS, low Disease Activity Score; MDAS, moderate Disease Activity Score; MOA, mechanism of action; MTX, methotrexate; RA, rheumatoid arthritis; RF, rheumatoid factor; SD, standard deviation; TNF, tumor necrosis factor

**Reference**

**World Health Organization:** Global Database on Body Mass Index. http://apps.who. int/bmi/index jsp?introPage=intro_3.html&
